# Coculture techniques for modeling retinal development and disease, and enabling regenerative medicine

**DOI:** 10.1002/sctm.20-0201

**Published:** 2020-08-07

**Authors:** Ali E. Ghareeb, Majlinda Lako, David H. Steel

**Affiliations:** ^1^ Sunderland Eye Infirmary, South Tyneside and Sunderland NHS Foundation Trust Sunderland UK; ^2^ Biosciences Institute, Newcastle University Newcastle‐upon‐Tyne UK

**Keywords:** biocompatible materials, cell transplantation, coculture techniques, microphysiological systems, organ culture techniques, organ‐on‐a‐chip, organoids, retina, retinal degeneration, tissue transplantation

## Abstract

Stem cell‐derived retinal organoids offer the opportunity to cure retinal degeneration of wide‐ranging etiology either through the study of in vitro models or the generation of tissue for transplantation. However, despite much work in animals and several human pilot studies, satisfactory therapies have not been developed. Two major challenges for retinal regenerative medicine are (a) physical cell‐cell interactions, which are critical to graft function, are not formed and (b) the host environment does not provide suitable queues for development. Several strategies offer to improve the delivery, integration, maturation, and functionality of cell transplantation. These include minimally invasive delivery, biocompatible material vehicles, retinal cell sheets, and optogenetics. Optimizing several variables in animal models is practically difficult, limited by anatomical and disease pathology which is often different to humans, and faces regulatory and ethical challenges. High‐throughput methods are needed to experimentally optimize these variables. Retinal organoids will be important to the success of these models. In their current state, they do not incorporate a representative retinal pigment epithelium (RPE)‐photoreceptor interface nor vascular elements, which influence the neural retina phenotype directly and are known to be dysfunctional in common retinal diseases such as age‐related macular degeneration. Advanced coculture techniques, which emulate the RPE‐photoreceptor and RPE‐Bruch's‐choriocapillaris interactions, can incorporate disease‐specific, human retinal organoids and overcome these drawbacks. Herein, we review retinal coculture models of the neural retina, RPE, and choriocapillaris. We delineate the scientific need for such systems in the study of retinal organogenesis, disease modeling, and the optimization of regenerative cell therapies for retinal degeneration.

## Graphical abstract

Neural retina, retinal pigment epithelium, and choriocapillaris are highly interdependent and therefore coculture techniques are required to understand normal development and disease. Furthermore, coculture techniques will enable the optimization of regenerative therapies and the development of transplantable multitissue sheets.

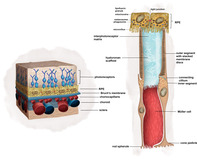

**Significance statement**

The light‐sensitive neural retina is nourished by the retinal pigment epithelium (RPE), while the choriocapillaris, a dense capillary network, supplies oxygen and metabolites. Coculture of these tissues is therefore required to understand normal retinal development and disease. Transplanted retinal precursors fail to fully integrate within host tissues and form the normal RPE‐photoreceptor and RPE‐choriocapillaris interactions which sustain vision. Coculture techniques will enable in vitro optimization of regenerative cell therapies for degenerative retinal diseases, forming a step to successful in vivo transplant experiments. Furthermore, coculture of neural retina, RPE, and choriocapillaris will facilitate the development of transplantable multitissue sheets.

## 1 INTRODUCTION

### 1.1 Retinogenesis

The finding that retinal homeobox (Rx)^+^ cells derived from ESCs and induced pluripotent stem cells (iPSCs) can self‐aggregate in low‐attachment culture to form retinal organoids with apical‐basal polarity, showed that neuroectodermal cells of the developing optic cup possess an inherent self‐organizing capacity.^1^, ^2^ This approach has created a new source of transplantable photoreceptor precursors and retinal sheets, which have been shown to integrate into degenerate retina forming synapses with host bipolar cells, responding to light and transmitting action potentials to the superior colliculus.^3^, ^4^, ^5^, ^6^, ^7^ The process of generating retinal organoids, however, remains costly and produces variable results.^8^ Furthermore, the transplantation of stem cell derived retinal tissue faces several challenges, which we will discuss.

**FIGURE 1:**
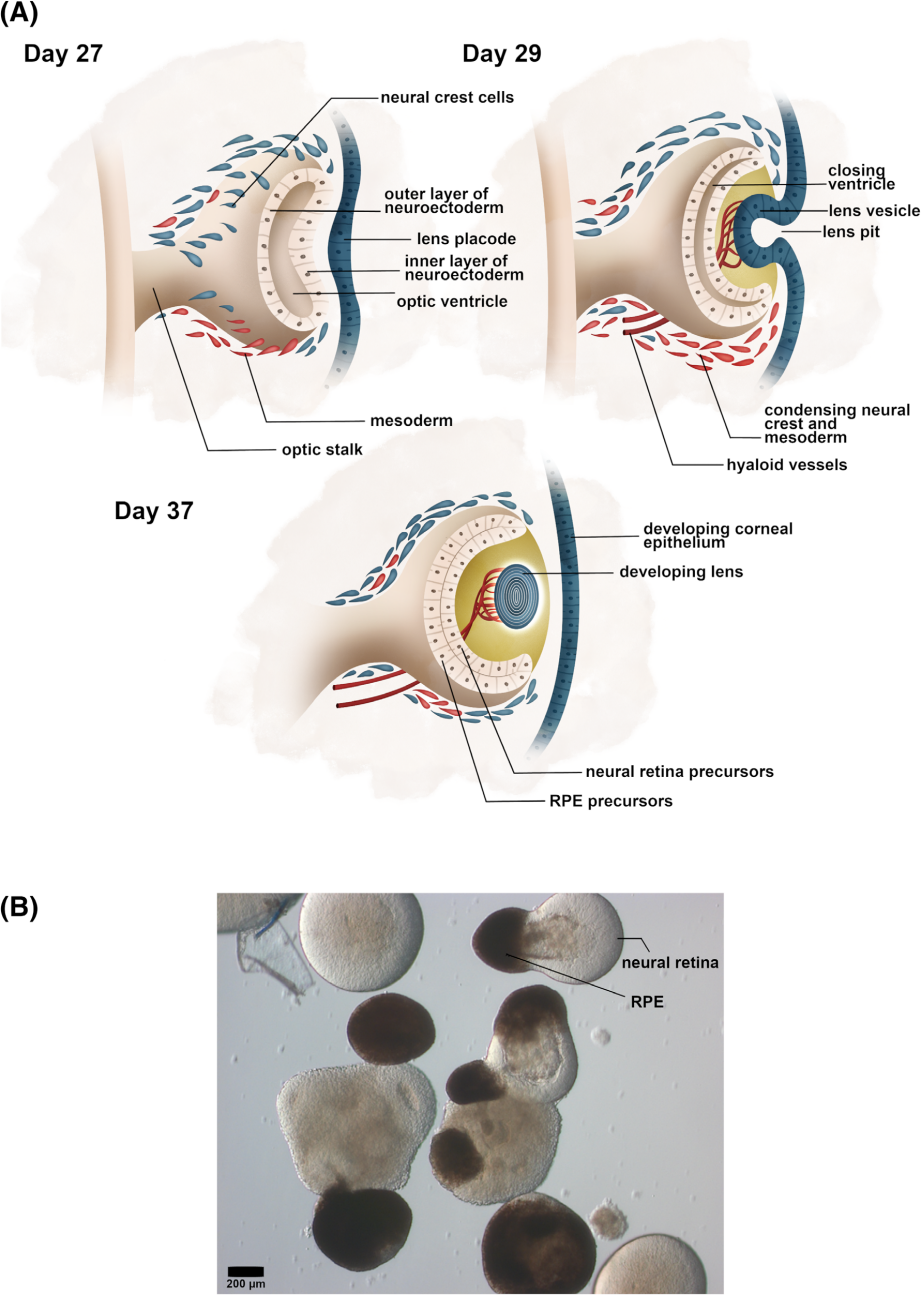
Understanding retinogenesis is critical to developing in vitro models and transplantable tissue. A, A para‐axial cross section is shown of the development of the optic vesicle and its invagination to form the bi‐layered, neuroectodermal optic cup which later develops into the adult RPE and neural retina (postconception days shown). At approximately day 37, the two layers of the optic cup become apposed and the optic ventricle is obliterated. Neural crest and mesoderm condense to form the choroid and choriocapillaris. Hyaloid vessels form the blood supply to the inner retina and their vitreous branches regress in later life. B, A bright field photograph of human iPSC‐derived retinal organoids at day 90 of differentiation. There is phase‐bright neural retina with photoreceptors orientated outward (not shown) and a single dark pole of RPE. Scale shown. iPSC, induced pluripotent stem cell; RPE, retinal pigment epithelium

Retinogenesis is orchestrated by a cascade of genetic master‐switches whose activation progressively narrows the developmental potential of precursors.^1^, ^2^, ^6^, ^9^ The developing RPE and neural retina are derived from a bi‐layered invagination of the neuroectodermal optic vesicle which is surrounded by tissues of mesenchymal, neural crest, and surface ectodermal origin during development (Figure 1A). The space between the two layers of neuroectoderm is obliterated in humans between postconception days 37 and 42 and this event physically unites the precursors of the RPE and the photoreceptors.^10^ It is after this event that the two layers differentiate into RPE and neural retina with the photoreceptor outer segments forming at the apical surface of the developing RPE and the RPE microvilli developing at the apical surface of the photoreceptor precursors.^10^ Thus, functional and physical maturation occurs in the context of physical cell‐cell contact. Outer segment formation occurs at approximately 112 days postconception and the RPE eventually phagocytoses photoreceptor outer segments to facilitate the recycling of visual pigments. Importantly, apposition of the developing RPE and neural retina precedes their maturation into functional retina.^10^, ^11^, ^12^

### 1.2 Synaptogenesis

Synaptogenesis in the developing human retina occurs sequentially from the developing fovea to the periphery, beginning with cones. The cone‐rich fovea provides high‐acuity color vision. Development of cone‐bipolar cell synapses begins in postconception weeks 8 to 10, before synaptic development of rods, and by week 13, all cones across the fovea express synaptic markers. However, core components of the synapse, the metabotropic glutamate receptor 6, and voltage‐dependent calcium channel α1.4 are not detected until fetal week 22.^13^ Rods are the predominant photoreceptor type and contact approximately 58 rod bipolar cells in the mature mouse retina. This convergence of rod afferent signals ensures high sensitivity of scotopic vision.^14^ Interestingly, mature mouse bipolar cells appears to retain useful plasticity, with the ability to extend dendrites to new target rods, while reducing the number of synapses containing multiple ribbons, in order to prevent oversaturation with input signals.^15^ Given the large number of cell types in the vertebrate retina, it is vital that postsynaptic target cells are correctly specified during development and remodeling. Specific pre‐ and postsynaptic membrane proteins linked by specific extracellular proteins that determine target type and are essential for correct synapse formation.^16^

### 1.3 Retinal organoids

Retinal organoids recapitulate the main events in the development of the mammalian retina both spatially and temporally and produce all seven cell types in a typical laminated fashion.^1^ Naturally, they have been used to study mammalian retinal development.^17^ Free‐floating embryoid bodies are generated from suspensions of, or free‐floating aggregates of, stem cells and then directed toward neural differentiation which is followed by eye field specification and maturation into retinal organoids. Recent research has focused on improving the efficiency of differentiation, developing disease models using patient iPSCs, testing gene therapies, staging the differentiation of retinal organoids, and single cell transcriptomics.^18^ Transcriptomic studies have characterized the molecular architecture of developing retinal organoids and correlated it with developing and adult human retina, creating large repositories of temporal expression data.^19^, ^20^ Recent work has studied the substantial variability between retinal organoid lines.^8^ Although the RPE‐photoreceptor relationship is indispensable to photoreceptor signal transduction and therefore vision, this relationship is not reproduced in retinal organoids, which often but not always, contain a pole of RPE oriented away from neural retina (Figure 1B). It is important to recall that the invagination of the optic vesicle during retinogenesis orientates the apical surfaces of the developing RPE and neural retina toward each other, a process which is incompletely recapitulated in retinal organoid development, possibly due to the absence of paracrine or tractional forces from surrounding tissues as occurs in vivo. Little work has been done on the coculture of retinal organoids with RPE,^21^ although it has been reported that the maturation of retinal organoid photoreceptors is faster in the presence of primary RPE in contact coculture.^22^

### 1.4 Choriocapillaris, Bruch's membrane, RPE, and neural retina form a single functional unit in vivo

**FIGURE 2:**
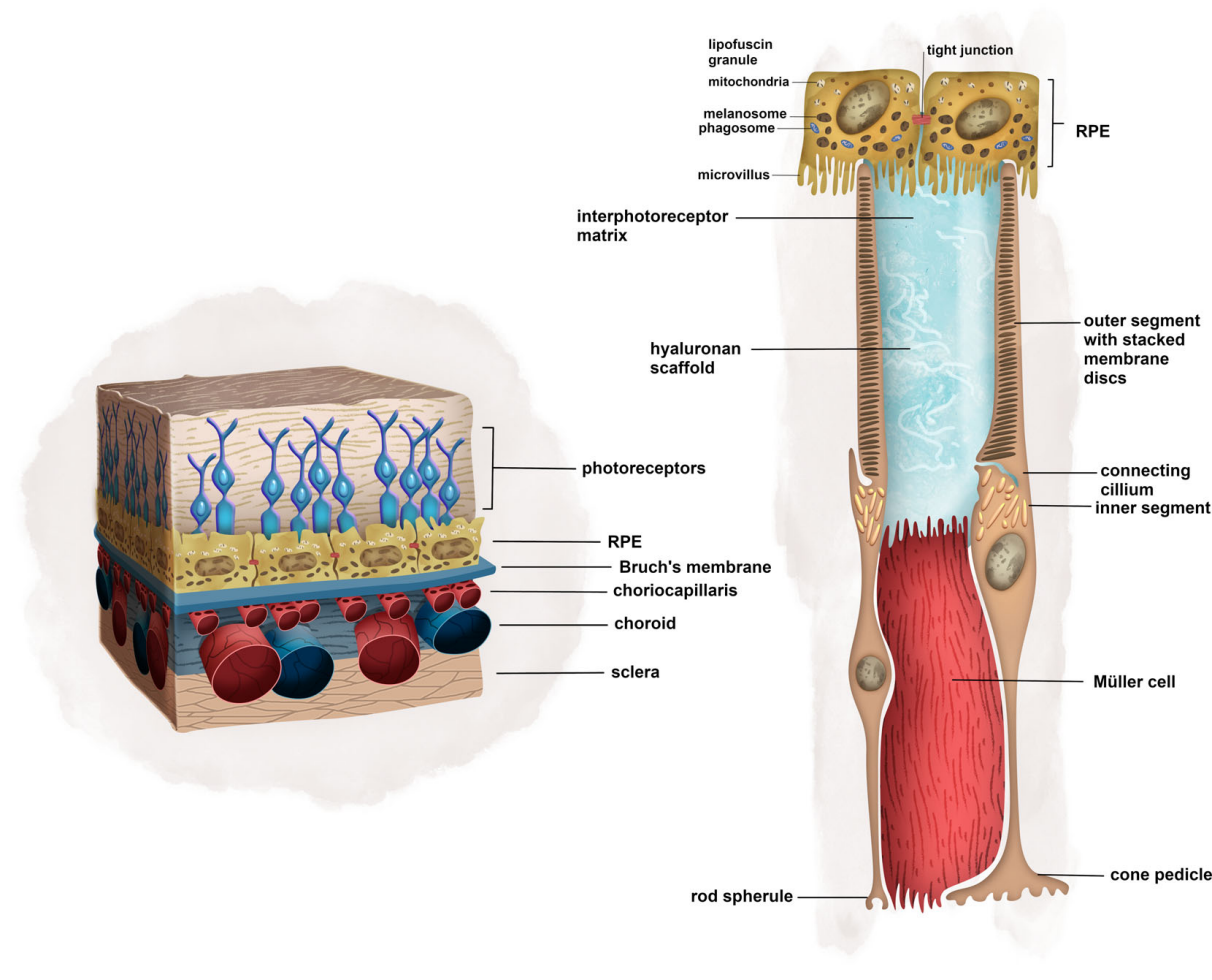
Illustration of the physical relationship between the neural retina, RPE, Bruch's membrane, and choroid, which form a single functional unit. The RPE cells phagocytose photoreceptor outer segments. The dense capillary network of the choriocapillaris is the blood supply to the highly metabolically active RPE and photoreceptors. RPE, retinal pigment epithelium

Photoreceptors are supported by growth factors from the RPE, which also phagocytose their distal outer segments, which is critical in the recycling of phototransduction pigments (Figure 2). Cone pigments are additionally recycled by Müller glia.^23^ Oxygen and metabolites are delivered directly to the inner neural retina by the retinal vasculature, branches of the central retinal artery—these are directly visualized on fundoscopy. However, it is the choriocapillaris, which meets the high metabolic demand of the RPE and outer neural retina including the outer nuclear layer (ONL).^23^ Distinct pathologies, which all have an endpoint of blindness, may have mechanisms which start in the choriocapillaris, RPE, or neural retina. At least 34 loci of genomic variants have been associated with age‐related macular degeneration (AMD), with diverse functions including lipid metabolism, immune function, and extracellular matrix (ECM) proteins.^24^ In one possible mechanism, AMD is believed to be initiated by complement dysregulation in the choriocapillaris, thereby leading to RPE loss and then photoreceptor degeneration.^25^, ^26^, ^27^, ^28^, ^29^ Although the exact sequence of events is a matter of active research, it is clear that all elements of the retina are eventually affected and areas of RPE and choriocapillaris degeneration colocalize.^25^, ^30^ Similarly, in inherited chorioretinal dystrophies of the choroid and RPE, photoreceptors are sequentially lost. This occurs in choroideremia, an X‐linked chorioretinal dystrophy caused by mutations in the gene Choroideremia (*CHM*), in which the earliest findings are loss of RPE seen on spectral domain‐optical coherence tomography. This is followed by photoreceptor degeneration around areas of RPE loss and loss of choriocapillaris density.^31^

Sandwiched between the RPE is a unique connective tissue sheet (Figure 2). Bruch's membrane is a five‐layered composite of RPE basement membrane, three elastin‐rich layers, and the choriocapillaris basement membrane.^32^ In adult life, it forms a barrier between the choriocapillaris and the RPE. The dense vessel network of the choriocapillaris is embedded within Bruch's membrane and unlike the larger choroidal vessels, develops in situ through a process called hemo‐vasculogenesis.^33^ One of the first pathological events in AMD is the formation of drusen and lipid deposition within Bruch's membrane, which holds prognostic significance for vision loss in AMD.^25^, ^32^, ^34^

In this review, we will first discuss the major challenges facing cell transplant for retinal regeneration, with a focus on transplant of neural retina, although this discussion and the use of coculture models is also relevant to those interested in RPE transplant. We then will discuss techniques used in the coculture of neural retina, RPE, and choriocapillaris and how these can be used to build better in vitro models of the retina. Finally, we will discuss the current state‐of‐the‐art in vitro models of the retina. Transplant of RPE patches has been investigated in phase I clinical trials and a phase I study using autologous iPSC‐derived RPE to treat geographic atrophy associated with AMD is now in the recruitment phase, although the technique is far from being optimized in preclinical models.^35^, ^36^ Although coculture is important to the optimization of RPE transplant, in this review we will use photoreceptor/neural retina transplant as a starting point for the discussion of retinal coculture. For reviews of RPE transplant, see Zarbin et al^37^ and Chichagova et al.^38^

## 2 STEM CELL REGENERATION OF THE RETINA: A CELL‐CELL INTERACTION PROBLEM

### 2.1 Transplant of dissociated photoreceptor precursors

The eye is an attractive target for regenerative cell therapies because of its relative immune privilege, accessibility, and the availability of advanced imaging technologies to noninvasively obtain high‐resolution images in vivo.^39^, ^40^

Initial studies using dissociated suspensions of photoreceptor precursors derived from early postnatal mice appeared to demonstrate maturation and functional integration in animal models of retinal degeneration.^41^, ^42^ However, it has become apparent that transfer of cytoplasmic material is the main mechanism of recovery of host retinal function (when no donor cells are present), regardless of whether primary cells or stem cell‐derived precursors are transplanted.^43^, ^44^, ^45^, ^46^ Given that the majority of cells in the host ONL (the layer containing photoreceptor nuclei) expressing donor cell markers are a result of cytoplasmic exchange, proving that dissociated photoreceptor precursors mature and integrate into the host retina by forming synapses with bipolar cells has been difficult.^43^, ^45^, ^46^ Others have reviewed this problem in detail.^47^, ^48^ Studies have shown some, albeit limited synaptic connection of photoreceptor precursors with host bipolar cells in rd1 mice where the ONL is absent and therefore cytoplasmic fusion not possible. These studies have however either not yet demonstrated visual improvement^6^, ^46^ or only modest visual improvement with survival of only a small fraction of the transplanted cells.^7^ Singh et al found that only a third of eyes had surviving donor precursors at week 12 and that an average of 7.9% of donor cells survived.^7^ In all cases, donor cells only partially matured without typical outer segment formation and no demonstrable interaction with the host RPE, which is critical to long‐term function.^6^, ^7^, ^46^

Recently, Garita‐Hernandez et al have circumvented the problem posed by the poor outer segment development and interaction with RPE in transplanted dissociated cells by transfecting primary mouse photoreceptor precursors and human iPSC‐derived photoreceptor precursors with bacterial opsins.^49^ However, much work needs to be done in selecting opsins with sensitivity over a useful range of the human visual spectrum and the material exchange paradigm will continue to be problematic for human‐human transplants.^49^

### 2.2 Transplant of retinal sheets

While long‐term survival and functional integration of dissociated photoreceptor precursors in the host ONL is unproven, studies using retinal sheets are able to point to long‐term graft survival in animals and humans, immunohistochemistry showing grafts maturing in the subretinal space and forming synapses with host bipolar cells many months after transplantation, and even visual recovery.^3^, ^4^, ^5^, ^50^, ^51^, ^52^, ^53^, ^54^, ^55^, ^56^, ^57^, ^58^ Foetal retinal sheet transplant in the fast‐degenerating *Rho‐S334ter line‐3A* rat shows detailed cortical responses to visual stimuli projected onto the graft area, providing a theoretical basis for retinal sheet transplant.^50^ Furthermore, transplanted foetal retinal sheet survived for 3 years in the subretinal space of a 94‐year‐old man with wet AMD, while microaggregates could no longer be detected in the same retina.^57^ Transplanted neonatal mouse microaggregates also show more maturation of outer segments than dissociated cells in the *rd1* mouse.^55^ Several experiments carried out in the 1990s provide data on the in vivo, long‐term survival of foetal retinal sheets in humans.^51^, ^52^, ^53^, ^54^ The long‐term survival of human iPSC retinal sheets in rat and primate models of retinal degeneration has been demonstrated (5 months and 2 years, respectively).^56^ The limited integration of interneurons from transplanted planar organoids has also been demonstrated.^59^ The available evidence therefore points to survival and likely functional engraftment of transplanted foetal/neonatal, ESC, and iPSC‐derived retinal sheets, while the available data do not currently demonstrate long‐term survival nor the exact functional abilities of integrated dissociated cells.

**FIGURE 3:**
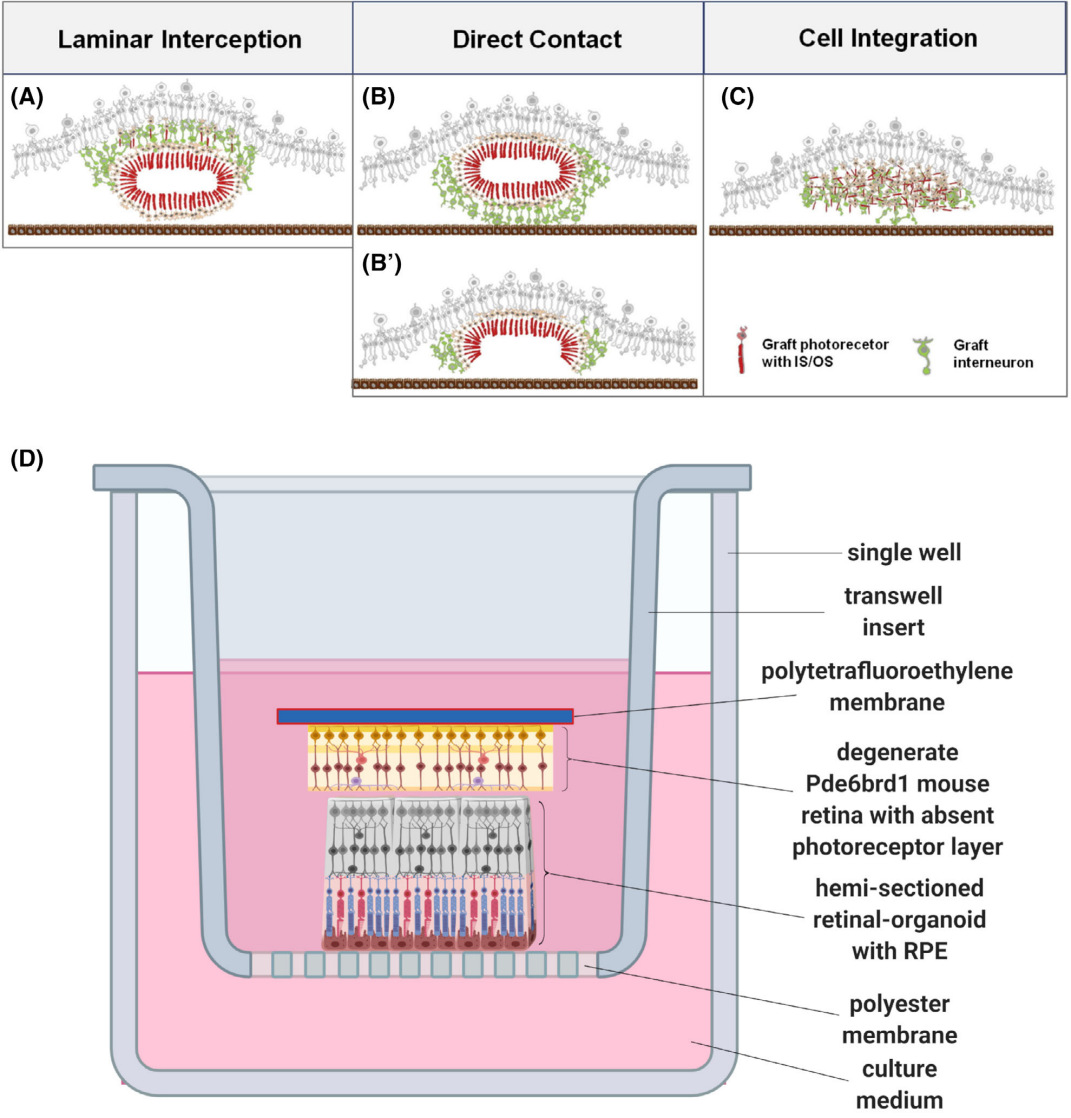
Rosette formation is common following transplant of stem cell‐ or foetal‐derived retinal sheets into the subretinal space. A, Graft INL prevents the direct contact of graft ONL and host INL, although some rare graft photoreceptors migrate toward the host INL and contact host bipolar cells. B, The graft ONL commonly integrated into the host INL; however, graft INL may lie between graft photoreceptors and host RPE, preventing their physical interaction. B′, Sometimes, incomplete rosettes allowed the graft ONL to integrate into the host INL with the graft ONL correctly oriented toward the host RPE. C, The graft laminar structure is sometimes lost following transplant and the graft photoreceptors migrate individually to the host INL. Reprinted from Assawachananont et al^5^ with permission from Elsevier. D, Illustration of a possible experimental setup allowing the in vitro optimization of stem‐cell derived retinal sheet transplant into a degenerate host retina. From the bottom, RPE could be cultured to maturity on a suitable membrane. Retinal organoids could be hemi‐sectioned to produce a flat sheet and placed photoreceptor‐side down onto the RPE. Degenerate retina from the Pde6Brd1 mouse, which lacks an ONL could be placed onto a suitable carrier membrane and placed on top of the hemi‐sectioned retinal organoid, thus recreating an in vitro subretinal space. For synapses to form between the Pde6Brd1 retina and the “transplanted” organoid, dendrites need to extend through the organoid inner retina (shown in gray scale). Ideally, the retinal organoid inner retinal layers, which may be redundant, could be ablated or dissected away before transplant. This experimental setup is inspired by Yanai et al.^60^ INL, inner nuclear layer; ONL, outer nuclear layer; RPE, retinal pigment epithelium

However, there are several challenges with retinal sheet transplant. In all animal models tested, and for both stem cell‐derived and primary cell grafts, anatomically correct integration of transplanted photoreceptors into the host ONL is currently hampered by rosette formation in the host (Figure 3A‐C).^3^, ^4^, ^5^, ^50^, ^61^ Rosette formation has two observable consequences: first, the transplanted inner nuclear layer (INL) obstructs the formation of synapses with the host bipolar cells (Figure 3A) (although bipolar cells have been observed to migrate or extend dendrites through the donor INL to make connections with donor photoreceptors).^4^ Second, the transplanted INL also prevents proper interaction of the host RPE with the donor photoreceptor outer segments, thereby precluding the phagocytosis of donor outer segment and the recycling of photopigments (Figure 3B).^3^, ^4^, ^5^, ^50^, ^56^, ^61^ The graft may also lose its laminar structure and graft photoreceptors may migrate individually toward the host INL (Figure 3C).^5^ Another obstacle (which is at least partially a consequence of the above) is that the formation of mature photoreceptors with complete outer segments has been highly variable in transplanted retinal sheets. The result of these three obstacles is that graft light‐sensitivity has not been robust and can sometimes only be measured after a period of dark‐adaptation.^3^, ^4^, ^56^ In their study of iPSC‐derived retinal sheets, Mandai et al supplied the grafted mice with intraperitoneal 9‐cis retinol in the belief that the grafts would not be able to isomerize all‐trans‐retinol due to the lack of contact with the host RPE.^4^

Optimizing the above factors is challenging owing to the difficulty in transplanting retinal sheets into live animals, the ethical and legal obstacles associated with animal research and the variability between animal hosts. Further work is needed to understand how the developmental potential of transplanted photoreceptor precursors is influenced by the environment of the degenerate adult retina. It is clear further queues are required to direct precursors toward generating mature cone and rod photoreceptors which form functional interactions with host RPE and INL neurons. Furthermore, if strategies such as optogenetics^49^ and ex vivo gene therapy and transplantation^62^ are used, in vitro optimization will be vital.

In vitro models of retinal degeneration which recapitulate the neural retina‐RPE interaction in disease states are needed. As we will now discuss, these models can be used to facilitate the faster optimization of regenerative cell therapies. To model the RPE more accurately in disease states, these models also require that other elements of the outer retina be present, namely Bruch's membrane and choroidal vasculature.

## 3 COCULTURE OF NEURAL RETINA AND RPE

Over the last 30 years, several efforts have been made to coculture RPE with neural retina to study retinal development, in vitro differentiation of stem cells and to develop regenerative cell therapies.

### 3.1 Models of differentiation and development

A key finding from experiments in which activation of a diphtheria toxin‐expressing gene selectively ablated the RPE of developing chick embryos was that the embryonic RPE is required for the development of other ocular tissues: transgenic chicks expressing the toxin showed anophthalmia, with the presence of only the extra‐ocular muscles and conjunctiva.^63^ Other studies have since shown that RPE‐derived trophic factors regulate mammalian neural retina development and that early RPE dysfunction during development alters the course of neural retina development, leading to neurodegeneration.^64^ The transcription factor icrophthalmia‐associated transcription factor (MITF) is highly expressed in RPE and other neural crest‐derived tissues, and mutations in this protein cause a variety of phenotypes in mice, most notably microphthalmia and retinal degeneration.^65^

Coculture models were created to study early retinal organogenesis, particularly the question of how apical‐basal polarity is attained. Embryonic chicken retinal cells can generate *organotypic* neural retina in vitro containing all cell layers. When cultured in isolation, embryonic retinal progenitors formed a back‐to‐front polarity of retinal lamination which was corrected when the cells were cocultured with pigmented cells from the ciliary region.^66^, ^67^ Sheedlo et al studied the effect of noncontact culture of primary rat RPE cells encased in semipermeable fibers with postnatal dissociated rat retina to understand the effects of RPE‐secreted trophic factors on retinal differentiation.^68^ They found an increase in opsin positive cells in the RPE coculture as compared to controls. Work from our own lab has found that culture of human induced pluripotent stem cells (hiPSC)‐ and hESC‐retinal organoids with RPE‐derived factors improves the generation of retinal organoids. Retinal organoids were cultured with a soluble solution of ECM peptides that was prepared by decellularizing freshly isolated bovine RPE (in practice this will also contain Bruch's membrane and choroid) and partially digesting the remaining matrix. A greater proportion of these retinal organoids express both RPE and neural retina (as opposed to RPE only or neural retina only). Furthermore, the expression of synaptic markers and light‐driven spiking activity recorded from retinal ganglion cells were improved. Media conditioned by hiPSC‐ and human embryonic stem cells (hESC)‐derived RPE enhanced the expression of rod‐specific markers and synaptic markers within retinal organoids.^69^

Other studies have used *organotypic* explant cultures to study mitogens secreted by the developing RPE^70^, ^71^ and to assess the differentiation potential of stem cells in vitro.^22^, ^72^ In an organotypic explant culture, the tissue architecture is preserved ex vivo. Fetal RPE secretes a different profile of trophic factors form adult RPE: higher concentrations of vascular endothelial growth factor‐A (VEGF‐A), brain‐derived neurotrophic factor, and pigment epithelium‐derived factor and significantly lower concentrations of leukemia inhibitory factor, basic fibroblast growth factor (bFGF), and nerve growth factor. This profile correlates with the ability of fetal RPE‐conditioned media to maintain porcine retinal explants in culture, with lower markers of cytotoxicity and apoptosis than in retinal explants cultured with adult RPE‐conditioned media. Furthermore, coculture of porcine retina with fetal RPE showed improved retinal survival over culture with fetal RPE‐conditioned media and higher levels of bFGF, heparin binding ‐ epidermal growth factor (HB‐EGF), and hepatocyte growth factor (HGF) were found in the culture media. The authors concluded that a synergistic interaction between the retinal explants and fetal RPE may account for the difference.^70^ Interestingly, another organotypic explant culture which used rat retina in direct apposition to RPE found that only hESC‐RPE could maintain the retinal explants for 2 weeks in culture with few TUNEL‐positive nuclei (an indicator of apoptotic DNA fragmentation), while ARPEJ and RPE19 failed to maintain retinal lamination and showed high numbers of TUNEL‐positive nuclei.^60^

On the other hand, the chick embryonic neural retina secretes factors which promote the maturation of the RPE.^73^ These factors increase the transepithelial resistance of RPE by directing the expression and localization of claudins.^74^ Interestingly, chick RPE (and human RPE)^75^ can attain a high level of differentiation in isolated in vitro cultures, expressing many RPE‐specific genes and showing a pigmented, cobblestone phenotype. However, full development of the RPE can only be attained with exposure to factors derived from the developing neural retina.^73^

Taken together, these models point to a mutualistic relationship between developing RPE and neural retina based on promoting tissue growth through paracrine signaling which is possibly complemented by signals generated through physical interaction.

### 3.2 Models for regenerative cell therapies

In vivo transplantation experiments must meet legal and ethical requirements and are expensive and time‐consuming. Furthermore, hypothesis testing is limited by interanimal variability unless large numbers of replicates can be made. Sheet transplants are especially difficult to test in animals owing to the need for bespoke delivery devices.^3^, ^4^, ^5^, ^50^, ^61^ In vitro models offer higher reproducibility at a lower cost which enables the optimization of cell therapies.^60^, ^76^, ^77^, ^78^ Additionally, human tissues can be derived from stem cells or fresh cadaveric tissue. Retinal organoids have proven utility in testing gene therapies and the CRISPR‐Cas9 system^79^; however, their utility in modeling the environment of the subretinal space is uncertain as the organoid photoreceptors and RPE are not correctly oriented. To adequately model the environment of the diseased subretinal space, retinal organoids may need to be combined with RPE and/or choriocapillaris. Ideally, these systems would allow access to the subretinal space and intravital monitoring of delivered stem cells without having to disturb the native subretinal environment. Figure 3D shows one possible conception of an in vitro system for optimizing transplant of retinal sheets into the degenerate *Pde6Brd1* mouse retina.^80^

Much previous work has used isolated neural retinal explants or RPE explants for studying organogenesis, differentiation of stem cells,^72^ or neuroprotective cell therapies.^78^, ^81^, ^82^, ^83^ These models have been reviewed elsewhere.^84^ Cocultured neural retina and RPE explants, arranged in an organotypic fashion allow functional interaction of the neural retina and RPE while allowing easy access to an artificial subretinal space for delivery of cell therapies,^60^, ^76^, ^77^, ^78^ gene therapies^85^ and potentially novel drugs.

An early characterization of a neural retina‐RPE explant coculture system designed for experimental testing of growth factors was by Caffé et al who explanted 210 neonatal mouse retinas, including 70 with neural retina only, 40 with neural retina and intact RPE, and 100 with neural retina, RPE and adjacent developing choroidal mesenchyme. After 21 days, electron microscopy showed those explants with adjacent RPE showed developed outer segment structure with regular disk stacks which were phagocytozed by adjacent RPE, although the outer segments were irregularly oriented. Mesenchyme developed into vascular structures ex vivo and appeared to improve the lamination of retina.^86^

Delivering cell therapies to in vitro models of the subretinal space may involve detaching the native retina from RPE. Kaempf et al characterized a coculture system whereby porcine neural retina and RPE/choroid were dissected apart and then immediately recombined. Compared to neural retina only, they found a large reduction in the level of apoptosis in the cocultured explants after 3 days ex vivo.^87^

A system designed for testing novel cell therapies came from the lab of Fernandez‐Bueno and Srivastava: porcine neural retina explants were cultured on the membrane of a tissue culture well insert, while primary porcine RPE were cultured on the bottom of the well.^77^ This allowed neural retina to be cultured at the medium‐air interface where there is a short diffusion distance for atmospheric oxygen, while at the same time allowing diffusion of RPE‐secreted mitogens from the bottom of the well. However, this was a noncontact culture with no physical interaction between the neural retina and RPE. The authors have further characterized their model and showed a marked reduction in glial fibrillary acidic protein (GFAP) expression in neural retina‐RPE cocultures vs neural retina alone, with preservation of photoreceptor inner segments and retinal architecture, while neural retina cultures alone showed rosette formation and profound reactive gliosis at day 9 ex vivo.^76^

Yanai et al developed an explant configuration specifically designed to experimentally optimize the integration of stem cell‐derived photoreceptor precursors which showed minimal cell death and good preservation of tissue architecture after 14 days of culture.^60^ Their experimental setup included the direct physical interaction of retinal explants and RPE just beneath the medium‐air interface, potentially facilitating a subretinal space environment more like the in vivo state (similar to Figure 3D). Retinal explants were derived from the fast‐degenerating S334ter rats and cadaveric human retinas. The authors compared the efficacy of hESC‐RPE, ARPE19, and RPE‐J to maintain rat and human retinal explants in culture. As noted earlier, they found that only hESC‐RPE was able to maintain neural retina in culture, while retinas cultured with AREPE19 and RPE‐J lost architecture and displayed many TUNEL positive nuclei by day 4 ex vivo. Similarly, neural retina explants cultured without RPE failed by day 4 ex vivo. Using this setup with hESC‐RPE, the authors were able to identify focal expression of synaptic markers BASSOON and RIBEYE in around 20% of transplanted photoreceptor precursors.^60^ It is not certain whether the increased survival of this model over that of Fernandez‐Bueno and Srivastava is due to the proximity of the RPE and neural retina explant, the use of hESC‐RPE, the inclusion of 2% B‐27 and 1% N‐2 in the explant culture or a variety of other possible factors. This and other studies^69^, ^70^ suggest that the milieu of growth factors secreted by RPE, and therefore its ability to maintain neural retina in culture, is differentiation‐stage dependent.

Cocultured explant models represent a promising model for in vitro optimization of regenerative cell therapies. However, there are several model parameters which can be optimized. Authors have pointed out that the time from death to enucleation is critical in determining the hypoxic damage to the neural retina.^81^ Fortunately, the retina is relatively resistant to hypoxia as compared to the brain and gives approximately a 20‐minute window before permanent damage (as measured by inability to restore a normal electroretinogram [ERG]).^88^ Liberation of the neural retina from the hypoxic globe during this window is therefore important.^81^ Anecdotally, avoiding killing test animals with CO_2_ and ideally aiming to enucleate under deep anesthesia could improve neural retina survival ex vivo. There is also evidence that replenishing glucose immediately postenucleation could help to minimize ischemic damage.^88^ Ideally, perfusion could be restored in ex vivo culture using microfluidic systems, allowing the constant replenishment of oxygen and nutrients.^89^

To date, ex vivo retina strategies have used surrogate markers of retinal function such as ONL thickness^90^ and immunohistochemical markers such as opsins and synaptic proteins^76^, ^91^ (quantified in terms of number of cells, area, or average intensity) or the presence of outer segment integrity.^76^, ^86^ More accurate data on functional enhancement of degenerate retinal explants could come from ERG assessments of retinal function which provide an assessment of the retinas ability to respond to light stimulation.^4^

## 4 COCULTURE OF RPE AND CHORIOCAPILLARIS

### 4.1 Models of differentiation and development

Work by Sakamoto et al demonstrated that primary bovine choroidal endothelial cells could form choriocapillaris‐like tubes in 3D‐culture comprised of type 1 collagen‐rich gel.^92^ The coculture of RPE overlying the 3D culture modulated the formation of capillary‐like structures by the endothelial cells more so than fibroblast and pericyte controls. Interestingly, overlying RPE promoted angiogenesis when coculture was from day 0 of culture but inhibited the growth of pre‐existing endothelial tubes when added at day 14. This underlies two important points: (a) RPE is apparently a hub, modulating multiple surrounding tissues and (b) tissue‐tissue interactions are complex with emergent properties only becoming apparent on accurate modeling of the interaction. The authors subsequently found that blocking bFGF and VEGF, but not transforming growth factor beta, inhibited RPE control over endothelial‐tube formation.^92^

In vitro models have shown that the RPE regulates angiogenesis in the choroid.^92^, ^93^, ^94^ This is in line with histopathological observations in rabbits with selective destruction of the RPE and with primary diseases of RPE, such as choroideremia which show that loss of choriocapillaris follows RPE loss.^31^, ^95^ Hamilton et al found that human umbilical vein endothelial cells (HUVECs) could be induced to a fenestrated choriocapillaris endothelial cell phenotype with expression of VE‐cadherin and ZO‐1 when cocultured with ARPE‐19 on opposite sides of amniotic membrane.^94^

Coculture experiments have also shown that there is a bidirectional relationship between RPE and choriocapillaris.^94^, ^96^ Hamilton et al also showed that the transfer of a 4kD dextran fluorescein tracer was almost totally inhibited by a HUVEC‐amnion‐RPE trilayer but not a HUVEC‐amnion‐corneal epithelial cell trilayer, and the amnion‐RPE bilayer alone was ineffective at inhibiting tracer transfer.^94^ Interestingly, experiments looking at the trans‐epithelial resistance in hESC‐RPE monocultures found that they are less than monocultures of human foetal RPE isolated from 16‐week‐gestation foetuses, even after the transepithelial electrical resistance (TER) is allowed to stabilize in a medium, which encourages fetal and hESC‐RPE maturation. Transcriptomic data showed that the expression of tight‐junction and membrane transport genes was reduced in hESC‐RPE.^97^ This suggests that RPE monoculture does not fully provide the queues for generation of a physiological outer blood‐retina‐barrier (oBRB), and this is common to both ARPE‐19 and hESC derived RPE.

Later, Spencer et al showed that ARPE‐19 phenotype is modulated by transwell coculture with HUVECs, with enhanced basement membrane deposition, phagocytic activity, ZO‐1 expression, and expression of visual cycle genes including RPE‐65.^96^ In the same year, Benedicto et al compared the choroidal endothelial transcriptional profiles of early postnatal mice, when the retina is undergoing terminal differentiation with adult mice with mature retinas. They isolated developing and mature choroidal endothelial cells, respectively, and found that the transcriptional programme of the developing choroid is focused on development and proliferation while adult choroidal endothelium is focused on extracellular matrix deposition and cellular adhesion.^98^ Using a transwell coculture system, they found that a variety of choroidal and nonchoroidal endothelial cell lines markedly enhance the transepithelial resistance of primary foetal RPE cultures, and that this effect is partially mediated by lysyl oxidases, a family of enzymes which catalyze elastin and collagen crosslinking. The formation of RPE tight junctions appeared to be dependent on the presence of stiff collagen anchor points in the basement membrane, the deposition of which is regulated by choroidal endothelium through yet unidentified paracrine factors. The presence of stiff collagen anchor points seems to be detected by β1‐integrin which modulates Rac1 and RhoA/ROCK pathways thereby regulating the RPE tight junctions.^98^ Together, these results suggest that the specific physical properties of Bruch's membrane direct immature RPE toward RPE maturation. These findings are important for the development of in vitro models as they suggest that an engineered biomaterial with the correct physical properties may be able to maintain a mature RPE phenotype at least partly *without* the need for cocultured choroidal endothelium.

### 4.2 Disease models—AMD

The oBRB is formed by the tight junctions between RPE cells (Figure 1). These cells are attached to their basement membrane which forms the innermost layer of Bruch's membrane, and whose outermost membrane is formed by the basement membrane of the choroidal endothelium.^32^ The degree to which these three layers are interdependent is critical to accurate modeling of disease and regenerative strategies. Models have investigated this question using cells from a variety of sources, either in contact coculture or separated by a Bruch's‐like substitute which may be synthetic or natural.

An important motivation for the development of such models is understanding the pathogenesis of AMD, one aspect of which is the formation of choroidal neovascular membranes and the dysregulation of complement pathways in the choriocapillaris where the pathology is thought to begin. Other motivations are ophthalmic drug discovery, studying pharmacodynamics and studying the physiological relationship between RPE, Bruch's membrane, and choroid and their development. Important questions for regenerative medicine are: *Is the inclusion of the choroid in in vitro models a prerequisite for accurate modeling of the RPE in health and disease*, *and do regenerative therapies need to also target the choroid in diseases such as AMD?* Other recent reviews have looked broadly at the current state of oBRB modelling.^38^, ^80^, ^99^, ^100^ Here, we will aim to answer the above questions, which are of relevance to the development of in vitro systems for testing regenerative therapies.

Most noncontact coculture models of the oBRB used a transwell insert culture system to separate the RPE from the endothelial cells.^96^ Models of pathogenesis have also used contact coculture (effectively a model of choroidal neovascularization—when choroidal endothelium invades through Bruch's membrane to the RPE), hypoxia or high doses of VEGF to model the disease phenotype, with mature primary endothelial cells and mature primary RPE.^96^, ^101^, ^102^, ^103^ The transwell model allows independent measurement of apical and basal secretion of growth factors, trans‐epithelial resistance, permeability to small molecules, and basement membrane deposition.^94^, ^96^, ^98^, ^101^, ^102^, ^103^ Some of these models can recapitulate complex disease processes. For example, Liu et al were able to assess reduced endothelial cell tube formation and invasion toward the RPE layer in a transwell coculture model.^101^ ARPE‐19 cells were cultured on polyethylene terephthalate transwell membranes with 8 μm pores, which allowed invasion of adjacent RF/6A endothelial cells cultured in Matrigel. Invading RF/6A endothelial cells on the RPE‐side of the membrane were fixed and counted. Using this assay, they studied the effect of RACK‐1 knockdown on an in vitro model of choroidal neovascularization.

Future work could use patient‐derived iPSCs to model the effect of high‐ and low‐risk AMD genotypes on cocultures of RPE and choriocapillaris.^26^, ^27^, ^29^ Such work may help to elucidate the early events which lead to AMD pathogenesis and help us to understand the mechanisms which lead to diverse clinical presentations in the AMD patient population.

## 5 BIOCOMPATIBLE MATERIALS FOR COCULTURE

The development of engineered biomaterials designed to mimic the interphotoreceptor matrix (IPM), Bruch's membrane, and choriocapillaris basement membrane will allow the development of improved coculture models and regenerative treatments, with improved differentiation efficiency, reduced phenotypic drift, improved survival in long‐term cultures, and improved lamination of in vitro or transplanted retina (reduced rosette formation). The use of biomaterials in in vitro models and retinal regeneration has been reviewed thoroughly.^89^, ^104^ Here, we will focus on their importance to coculture models of the retina.

It is now clear that specific physical properties of the extra‐cellular environment are critical signals which direct embryonic and tissue stem cell differentiation. These include both cell‐cell and cell‐ECM contacts.^105^, ^106^, ^107^, ^108^, ^109^, ^110^, ^111^ Physical queues alone can direct tissue‐specific differentiation and can replace some of the factors required to maintain pluripotency in vitro. Substrate stiffness has been identified as a critical environmental queue in maintenance of pluripotency and lineage specification.^107^, ^110^, ^112^, ^113^ The presence of specific ECM proteins facilitates tethering to biological or synthetic substrates and thereby facilitates efficient mechano‐transduction of substrate stiffness to intracellular signals.^109^, ^111^, ^112^, ^114^ Oligodendrocyte precursor cells are multipotent cells responsible for regeneration in the central nervous system. It has recently been shown that age‐dependent loss of function in oligodendrocyte precursor cells can be recapitulated by culture on stiff hydrogels which mimic the aged connective tissue and this loss of function can be restored on soft hydrogels which mimic young brains.^110^ Similarly, investigators have already shown that cultured RPE has reduced adhesion and survival on damaged or aged RPE.^115^, ^116^ Bruch's membrane is known to stiffen with age and cultured RPE show reduced phagocytic capacity on stiff vs soft scaffolds.^117^, ^118^ Resurfacing of aged Bruch's with ECM components can improve RPE survival in vitro,^119^ which may point to loss of anchoring points and/or changes in stiffness as important pathological mechanisms. Our group has shown that various laminins are expressed throughout the developing human retina including Bruch's, and that the distribution and expression of these laminins changes in concert with remodeling and lamination of the developing retina around weeks 10 to 12 postconception. Furthermore, these expression patterns were conserved in retinal organoids.^120^ Changing physical queues in the developing retinal environment is possibly a mechanism by which retinogenesis is coordinated.

Most attempts at transplant of outer retinal tissues have focused on the transplant of dissociated photoreceptor precursors.^47^ Furthermore, most published organoid culture involves free‐floating culture of the optic vesicles with no surrounding supportive ECM which is known to be critical for proper RPE maturation.^18^, ^98^ It is possible that this partially underlies the current deficiencies in retinal organoid architecture, and indeed it has been shown that both hydrogel and complex ECMs can improve organoid development.^69^, ^121^ Singh et al generated transplantable planar organoids by culturing them on a biomimetic and biodegradable scaffold.^59^ Both in vitro models of the retina and regenerative cell therapies could benefit from the application of rationally designed biomaterials.

### 5.1 Biosynthetic Bruch's membrane

As RPE and choroid endothelium are known to interact synergistically,^92^, ^93^, ^94^, ^96^, ^98^ development of choroid‐RPE cocultures is an attractive option both for in vitro modeling and for transplantation.^122^

The ideal rationally designed Bruch's membrane would have a permeability sufficient to allow metabolite diffusion between cell layers while maintaining structural integrity to physically separate choroidal endothelium and RPE.^123^ It would allow cell tethering to its surface while having a stiffness which could be physically tuned to the optimum for RPE on one side and choriocapillaris on the other. It would biodegrade over a sufficient period to allow RPE and choriocapillaris to lay down basement membrane.^124^ The specific complement of ECM proteins may also be important and can be recapitulated using decellularized ex vivo Bruch's^125^ or decellularized choroidal ECM which can be revascularized with endothelial cells.^126^ Ex vivo Bruch's membrane from individuals with outer retinal disease may serve in coculture models of these diseases.^127^

Other physical properties which have been investigated are porosity, wettability, and ion diffusion capacity in a variety of synthetic and natural explants (reviewed in detail here)^128^ and using a variety of surface coating to facilitate cell adhesion^122^, ^123^, ^129^, ^130^; however, these studies have not investigated stiffness or tethering properties. Unsurprisingly, several polymers and several surface coatings containing ECM proteins such as collagens and laminins were found to be suitable for culture of hESC‐derived RPE, although the identification of specific conditions which optimize RPE function have been equivocal.^104^, ^128^, ^129^ Other studies have focused on the protein composition of the RPE‐secreted matrix but not on its physical properties.^125^, ^131^ Few studies have specifically examined the effect of scaffold stiffness on RPE culture and more work is required to identify the optimal tethering and stiffness properties which will facilitate cocultures models of the outer retina.^117^, ^131^, ^132^, ^133^

Poly lactic‐co‐glycolic acid is a popular biomaterial for drug delivery and tissue engineering, and is currently being investigated as a vehicle for the subretinal delivery of iPSC‐derived RPE to treat AMD.^36^, ^124^ Its advantages include the availability of fabrication methods, biocompatibility, biodegradability, and tunable mechanical properties.^134^

For the transplant of retinal sheets, or perhaps sheets containing neural retina in addition to RPE and/or choroid, scaffolds which maintain cellular packing density and cellular orientation are desirable. The use of two‐photon polymerization has been used to generate scaffolds with submicron features. Retinal progenitors nested within 25 μm pores oriented themselves perpendicular to the scaffold.^135^

### 5.2 Biosynthetic IPM

While photoreceptor soma are supported physically and physiologically by Müller cells, their outer segments are embedded in a highly specialized extra‐cellular matrix, the IPM (Figure 1). The IPM is a highly complex hyaluronan matrix consisting of regularly repeating units of peanut agglutinin and wheat germ agglutinin which respectively sheath cone and rod outer segments.^136^ Photoreceptor outer segments are also closely associated with hyalectans (Versican and Brevican) and Interphotoreceptor Matrix Proteoglycans (IMPG1 and IMPG2), which interact with the hyaluronan scaffold.^137^ Photoreceptor outer segments and RPE do not form intercellular connections and therefore the IPM may provide an adhesive interface.^138^ The IPM additionally facilitates transport of signaling molecules and metabolites between the RPE and photoreceptors. Its components undergo light‐dependent conformational changes, underlying its importance in maintaining efficient phototransduction.^139^ Major structural components, such as hyaluronan, are secreted by the RPE while other protein components such as interphotoreceptor retinoid binding protein are secreted by the photoreceptors.^138^ Our work has shown that retinal organoids express several components of the IPM and that the expression of IPM components IMPG1 and CD44 in retinal organoids is required for proper development of outer segments and structuring of the IPM.^137^

Biosynthetic IPM may be useful in encouraging host RPE interaction and reducing rosette formation in the case of stem cell‐derived neural retina transplant. Hyaluronic acid‐based hydrogels among several other biomaterials have been investigated as a vehicle for cell therapies in the retina and central nervous system.^140^ Work from our group has shown enhanced differentiation of retinal organoids cultured with RGD‐alginate hydrogels.^69^ Re‐engineering the complex structure of the IPM would be an enormous task. Presumably, in vitro cocultures of neural retina and RPE would be capable of generating an IPM although this needs to be determined. In this case, culturing RPE on a Bruch's‐like membrane could be enough to allow cultured RPE to begin generating IPM. Masaeli et al have attempted this strategy and bioprinted primary porcine photoreceptors onto ARPE19 cultured on a sheet of gelatine methacrylate.^141^

## 6 MICROPHYSIOLOGICAL SYSTEMS

Microphysiological systems (organ‐on‐a‐chip) combine in vitro coculture models with biomaterials and/or microfluidics in order to recapitulate one or more key functions of a tissue.^142^, ^143^ They offer control over metabolite/drug delivery and waste removal, compartmentalization, simulation of flow and movement, and real time physical measurements. There are numerous examples of microphysiological systems outside of ophthalmology. These strategies increase the complexity of in vitro models but must focus on reproducing a specific tissue function or process in order to remain interpretable and practically feasible. Identifying the current barriers to effective stem cell‐derived retinal transplant and modeling them individually will aid progress in the field of retinal regenerative therapies. As we have previously identified, these barriers include efficient synaptic integration of the transplant, interaction between photoreceptors and RPE and maturation of transplants to a fully functional form, among other issues.^144^

One focus has been to “upgrade” ex vivo models and retinal organoids. By introducing microfluidics, it is possible to achieve a more physiological steady state in terms of removing metabolic by‐products and introducing nutrients and oxygen. This could help to increase the longevity of retinal explants.

Retinal organoids suffer from a necrosis of centrally located cells likely due to the diffusion limit of oxygen. Recent characterization of cerebral cortical organoids has found that maturation into cortical subtypes is impaired by the activation of stress pathways. This activation is ameliorated, and subtype specification improved by transplantation of cortical organoids into the mouse cerebral cortex. Likewise, primary progenitors transplanted into cortical organoids adopt stress pathway activation.^145^ This indicates it is the in vitro environment which impairs cortical organoid maturation.

Microphysiological systems may be used to investigate local effects of cell therapy, for example, on glial activation,^146^ to study the regeneration of the oBRB in the case of stem cell‐derived RPE transplant,^100^ to optimize regenerative therapies in disease‐specific states (eg, wet AMD)^100^ and to optimize synaptic integration of transplanted neural retina.^147^ Su et al observed synaptogenesis between two populations of developing mouse retinal precursors in 4 μm guiding channels.^147^ Compartmentalized experimental setups such as this could be used to optimize synaptogenesis between stem cell derived retina and host neurons.

**FIGURE 4:**
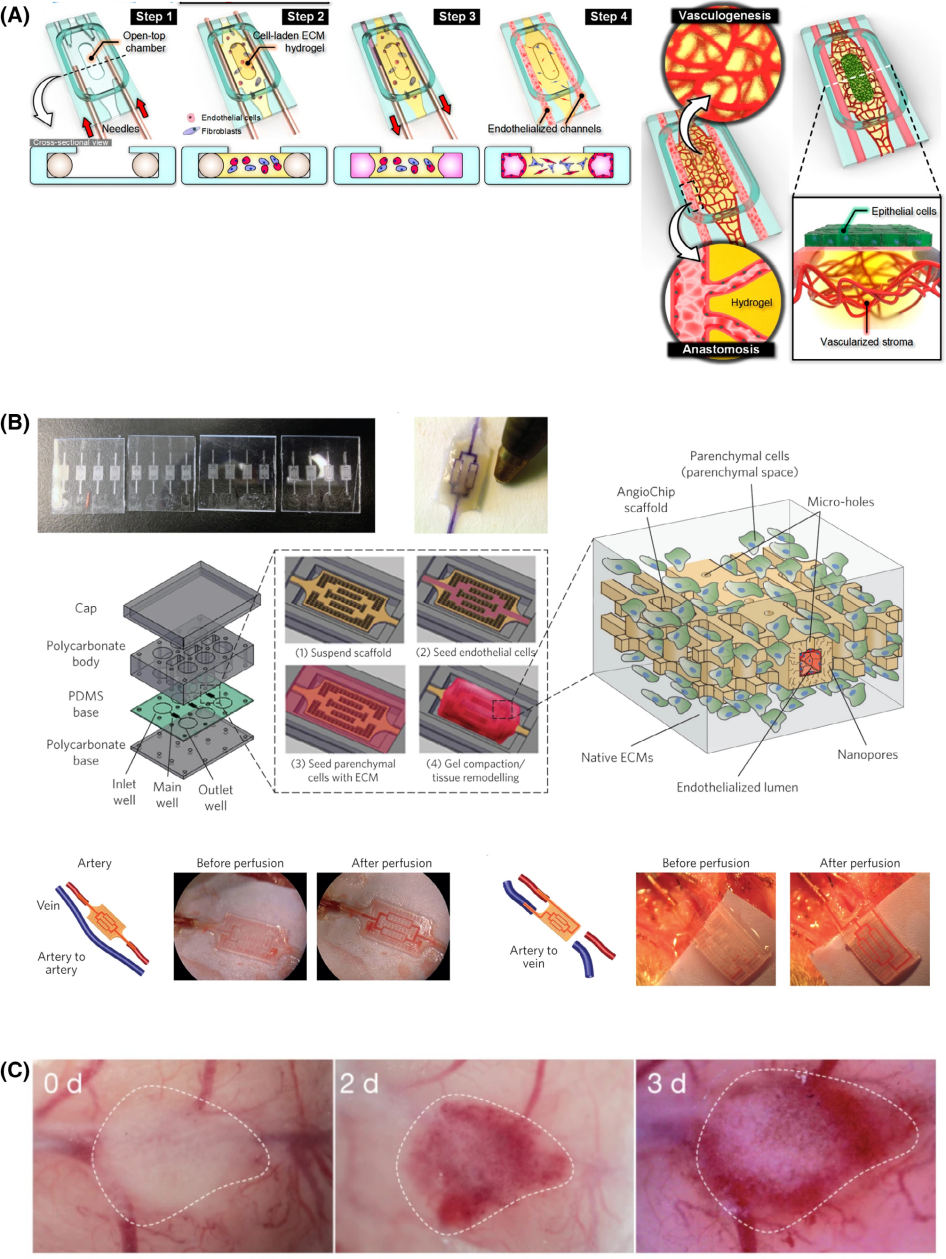
Perfusable in vitro generated vasculature may provide models of choriocapillaris. A, De novo generation of endothelial networks within an extracellular matrix‐laden hydrogel. These networks anastomose with main channels (representing arteries and veins, respectively) and the endothelial network can be perfused with tissue culture medium. RPE is cultured over the vascular networks to generate a model of the RPE and choriocapillaris. Reprinted with permission from Paek et al.^148^ Copyright 2019 American Chemical Society. B, Synthetic microvascular networks embedded within a hydrogel become lined with endothelium and allow remodeling of the surrounding hydrogel by parenchymal cells. In vitro perfusable tissue is thereby generated and can be linked to a host blood supply via surgical anastomoses (in this case, the rat femoral artery and vein). Adapted by permission from Springer Nature Customer Service Centre GmbH: Zhang et al.^149^ Copyright 2016. C, Liver organ buds generated by recapitulation of mesenchymal condensation are perfused by the host vasculature when transplanted into the mouse cerebrum (days post‐transplant shown). Adapted by permission from Springer Nature Customer Service Centre GmbH: Takebe et al.^150^ Copyright 2013 Springer Nature. RPE, retinal pigment epithelium

Paek et al designed a polydimethylsiloxane (PDMS) culture chamber, which they filled with primary choroidal endothelium and choroidal fibroblasts laden in a fibrin/type 1 collagen hydrogel. They observed self‐assembly of endothelial vascular networks which anastomosed with an input‐output circuit and were thereby perfused with culture media (Figure 4A). They cultured iPSC‐derived RPE on this hydrogel and found greater basement membrane deposition, melanosome expression, and RPE‐65 expression in their coculture model vs monocultures.^148^ Interestingly, they were also able to create a vascularized solid tumor‐on‐a‐chip model in which the self‐assembled vascular networks anastomosed with the tumor blood supply.^148^ Another strategy for in vitro vascular networks is shown in Figure 4B.^149^ This raises the possibility of perfused retinal organoids in an adapted microphysiological system as has been achieved with liver organoids (Figure 4C).^150^, ^151^

Achberger et al have created the most advanced retinal microphysiological system to date by coculturing 180‐day‐old human iPSC retinal organoids in individual wells, embedded in a hyaluronic acid‐based hydrogel over a layer of human iPSC RPE.^152^ They observed enhanced maturation of photoreceptor outer segments and importantly, RPE phagocytosis of photoreceptor outer segments. Although the retinal organoids were far from adult retina in terms of maturation, and the choroid was not represented, this project marks a clear advance. Retinal organoids were embedded in the chip at day 180 of differentiation, while in vivo human neural retina and RPE develop in close contact from approximately day 30.^10^ Recapitulation of human retinogenesis may improve the RPE‐photoreceptor interaction, speedup maturation and enable in vitro modeling of retinogenesis. Furthermore, this model does not overcome the problem of necrosis at the organoid center due to the oxygen diffusion limit as the organoids are not vascularized.^152^

## 7 CONCLUSIONS

Coculture models will advance our understanding of outer retinal physiology and disease. Furthermore, they will help optimize the delivery, integration, maturation, and functionality of stem cell‐derived retina for transplantation.

In our search, we have not identified a coculture system which incorporates neural retina, RPE, and choriocapillaris. As we have shown, all the techniques to achieve such a system are available. In the coculture systems we have reviewed, the neural retina is most frequently represented by retinal explants, which can be taken from animals with retinal degenerations. However, the neural retina component of coculture models may also be represented by hESC‐ or iPSC‐retinal organoids. In this way, human retinal organogenesis and patient‐specific disease models can be studied.

Microphysiological systems (retina‐on‐a‐chip) incorporate biomaterials to mimic Bruch's membrane, choroidal basement membrane, or the IPM and culture tissues in a 3D organotypic fashion. They may also incorporate in vitro generated and perfusable vascular networks to represent the choriocapillaris. Future work may look at incorporating lab‐on‐a‐chip technology to allow the real‐time measurement of calcium dynamics, cell trafficking, or light responses, for example.

These in vitro models need only capture the essential elements of complex in vivo interactions to reproduce phenomena of interest. In vitro models for optimization of neural retina transplant may benefit from focusing on recapitulating the environment of the host subretinal space, the important elements of which may be the neural retina, RPE, and IPM. Models for the optimization of RPE transplant may focus on modeling diseased Bruch's membrane and choriocapillaris.

Future work may also examine the optimum way to integrate retinal organoids into coculture with RPE and/or choriocapillaris. Factors such as day of differentiation, coculture media components, and RPE lines need to be optimized. Additionally, biomaterials can play an important role in recapitulating the retinal environment. Although much work has been done in identifying the building blocks of retinal ECM, more work needs to be done in identifying the 3D organization of tethering domains and physical properties which emulate the healthy or diseased retinal ECM.

Recapitulating embryogenesis by providing the correct developmental queues will direct stem cells toward developing in vitro models and tissues suitable for transplant. A significant barrier to successful cell therapies may be providing immature transplants with the correct cues for integration into an adult diseased host environment. Advance in vitro coculture models will be invaluable tools in solving this problem.

In combination with retinal organoid technology, these strategies will lead to realistic, disease‐specific models of ocular diseases. Using these models, we will be able to create *rationally designed* stem cell therapies for the retina and iteratively improve these therapies before moving to in vivo models and human trials.

## CONFLICT OF INTEREST

D.H.S. declared consultant/advisory role Alcon, Roche, Gyroscope and research funding from Alcon, Bayer. The other authors declared no potential conflicts of interest.

## AUTHOR CONTRIBUTIONS

A.E.G.: collection and/or assembly of data, data analysis and interpretation, manuscript writing; M.L., D.H.S.: conception and design, contributed to manuscript writing, final approval of manuscript.

## Data Availability

Data sharing is not applicable to this article as no new data were created or analyzed in this study.
